# Magnetic Hydroxyapatite Composite Nanoparticles for Augmented Differentiation of MC3T3-E1 Cells for Bone Tissue Engineering

**DOI:** 10.3390/md21020085

**Published:** 2023-01-25

**Authors:** Vignesh Krishnamoorthi Kaliannagounder, Mohammad Amjad Hossain, Jong-Hoon Kim, Muthukumar Thangavelu, Aravinthan Adithan

**Affiliations:** 1 Department of Bionanosystem Engineering, Graduate School, Jeonbuk National University, Jeonju 54896, Republic of Korea; 2 Division of Mechanical Design Engineering, Jeonbuk National University, Jeonju 54896, Republic of Korea; 3 College of Veterinary Medicine, Biosafety Research Institute, Jeonbuk National University, Iksan 54896, Republic of Korea; 4 Department of BIN Convergence Technology, Department of Polymer Nano Science & Technology and Polymer Materials Fusion Research Center, Jeonbuk National University, Deokjin-gu, Jeonju 54896, Republic of Korea; 5 Department of Molecular Pharmacology and Physiology, Morsani College of Medicine, University of South Florida, Tampa, FL 33612, USA

**Keywords:** hydroxyapatite, magnetic nanoparticles, MC3T3-E1 cells, magnetic hydroxyapatite, osteogenic differentiation

## Abstract

Progressive aging harms bone tissue structure and function and, thus, requires effective therapies focusing on permanent tissue regeneration rather than partial cure, beginning with regenerative medicine. Due to advances in tissue engineering, stimulating osteogenesis with biomimetic nanoparticles to create a regenerative niche has gained attention for its efficacy and cost-effectiveness. In particular, hydroxyapatite (HAP, Ca_10_(PO_4_)_6_(OH)_2_) has gained significant interest in orthopedic applications as a major inorganic mineral of native bone. Recently, magnetic nanoparticles (MNPs) have also been noted for their multifunctional potential for hyperthermia, MRI contrast agents, drug delivery, and mechanosensitive receptor manipulation to induce cell differentiation, etc. Thus, the present study synthesizes HAP-decorated MNPs (MHAP NPs) via the wet chemical co-precipitation method. Synthesized MHAP NPs were evaluated against the preosteoblast MC3T3-E1 cells towards concentration-dependent cytotoxicity, proliferation, morphology staining, ROS generation, and osteogenic differentiation. The result evidenced that MHAP NPs concentration up to 10 µg/mL was non-toxic even with the time-dependent proliferation studies. As nanoparticle concentration increased, FACS apoptosis assay and ROS data showed a significant rise in apoptosis and ROS generation. The MC3T3-E1 cells cocultured with 5 µg/mL MHAP NPs showed significant osteogenic differentiation potential. Thus, MHAP NPs synthesized with simple wet chemistry could be employed in bone regenerative therapy.

## 1. Introduction

Of the emerging fields in medical science, regenerative medicine is one that has evolved recently. The stability of an organism depends on the bone, and it also helps in several functions in the human body [1]. Although bone can regenerate itself, which is adequate for small cracks or minor damage, there is a chance that with aging, the structural and functional efficiency of the bone tissues will be altered [2,3] and could lead to osteoporosis, which exhibits a decrease in bone mass and quality and thus leads to osteoporotic fracture [4,5]. So far, the clinically available treatments for these diseases utilize autografts (osteoinductive) and allografts (osteoconductive) [6,7,8]. However, using these grafts may lead to challenges such as reduced bioactivity, inflammation, lack of availability, additional surgery, unsuitable shape and size, and donor-site injury [9,10]. These limitations can be tackled by developing novel biomaterials to repair and regenerate damaged tissues [4]. The most common biomaterials for bone tissue engineering are bioceramics, polymers, metals, and composite materials. Bioceramics are a type of inorganic biomaterial that has been widely employed in bone treatment due to their compositional similarities to human bone, high compressive modulus, biocompatibility, and ability to transfer bioactive ions [2,11].

In the bone extracellular matrix (ECM) component, the major inorganic material, which comprises approximately 60%, is composed of calcium phosphate minerals in the form of hydroxyapatite (HAP; Ca_10_(PO_4_)_6_(OH)_2_) [12,13]. Therefore, numerous studies have utilized synthetic bioceramics composted with calcium phosphate minerals such as hydroxyapatite, tricalcium phosphate, and whitlockite or their composite scaffolds to partially mimic native inorganic bone regenerative microenvironment to achieve tissue mineralization, integrity, and recruited stem cell regulation for bone turnover [14,15,16].

Recently, many researchers have used scallop shells to synthesize various inorganic materials such as calcium oxide (CaO) [17], calcium hydroxide (Ca(OH)_2_) [18], and HAP [19]. Takahashi et al. reported that there is about 98–99% calcium carbonate, trace inorganic materials, and 1–2% organic content available in scallop shells [20]. The recent study also reports that the scallop-shell-derived, calcium-containing materials are used as catalysts [21] and antibacterial reagents [22,23]. Among those calcium phosphate bioceramics, HAP and nano-HAP have been widely utilized for bone and dental implants due to their hexagonal crystal structure, similar to the mineral structure of bone; excellent biocompatibility; high mechanical strength; bioactivity; and osteoconductivity. Furthermore, HAP and nano-HAP are the most stable forms of calcium phosphate minerals with low solubility in physiological environments [24,25,26,27]. Although hydroxyapatite has a strong pro-remineralization impact, it is still limited and cannot replace natural remineralization. A relevant study has also revealed that crystal hydroxyapatite may not be capable of totally replacing natural bone [28]. To overcome this issue, ions may thus be doped or integrated to improve the bioactivity of this material. Recently, it has been reported that incorporating metallic ion nanoparticles can further enhance osteogenic properties because they are natural elements in the tissues that participate in bone metabolism. Moreover, these ions can regulate cell behavior, are stable, non-toxic, and feasible to track using magnetic resonance imaging (MRI). This ion-integrated composite scaffold is a promising trend in bone tissue engineering. Some examples of such ions are Zn, Mg, Fe, (Zn-Ni) ferrite, Na, Sr, Si, etc. [29,30,31].

Among transition metals, iron (Fe) ions could potentially enhance mechanical strength and provide magnetic properties to the bone scaffolds, which in turn could stimulate stem cell signaling pathways. El-Meliegy et al. reported that the Fe_2_O_3_ doping enhanced the bending strength of bioglass (BG) by forming the octahedral coordination with BG structural network and evidenced better bioactivity and comparatively acceptable cell viability for Fe-doped scaffolds [32]. Recently, magnetic iron oxide nanoparticles (MNPs) have been widely employed in bone tissue engineering due to their high coercivity, magnetic susceptibility, and controllability [33]. Wu et al. evaluated the synergistic effect of MNPs and exosomes derived from bone mesenchymal stem cells (BMSC-Exos) by applying with and without a static magnetic field (SMF) and demonstrated superior osteogenesis and angiogenesis by targeted regulation of exosomal miR-1260 [34]. In terms of promoting osteogenic differentiation, the addition of superparamagnetic MNPs aids in controlled growth factor delivery using a magnetic field, regulation of signaling pathways, protein adsorption by using SMF, and mild magnetic hyperthermia with an alternating magnetic field (AFM) [35,36,37]. Therefore, in this study, magnetic iron oxide decorated hydroxyapatite nanoparticles (MHAP NPs) were prepared by the wet chemical co-precipitation method and characterized by using field emission scanning electron microscopy (FESEM), bio-transmission electron microscopy (BioTEM), energy dispersive X-ray Spectroscopy (EDS) elemental mapping, X-ray diffraction (XRD), and Fourier transform infrared (FT-IR) spectroscopy. Invitro studies were performed using the mouse calvaria-derived osteogenic progenitor MC3T3-E1 cell line, which could only differentiate into osteoblast [38]. Thus, the MHAP NPs were evaluated against MC3T3-E1 cells for toxicity, ROS activity, proliferation, and osteogenic differentiation potentials.

## 2. Results and Discussion

### 2.1. Physicochemical Characterization of Nanoparticles

**Figure 1:**
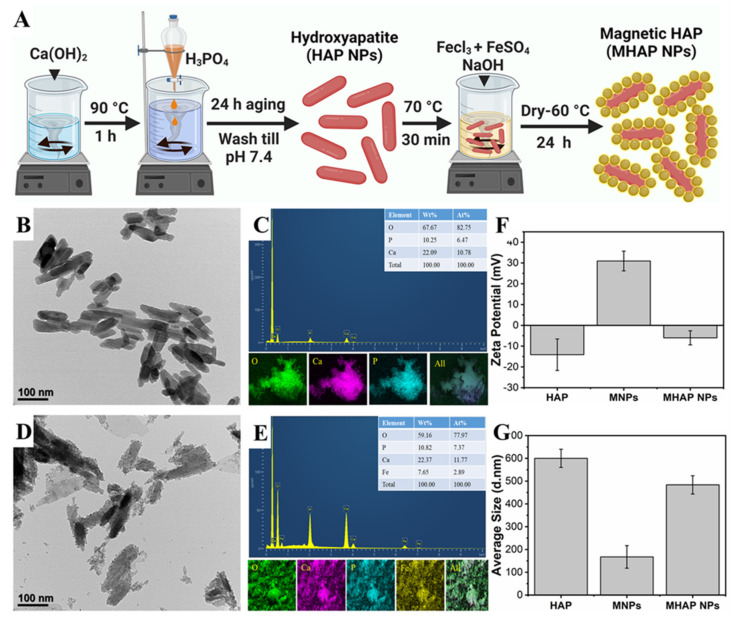
**(A**) Schematic illustration of HAP and MHAP NPs synthesis. (**B**,**D**) BioTEM images of HAP and MHAP NPs, respectively. (**C**,**E**) EDS and EDAX mapping of HAP and MHAP NPs, respectively. (**F**) Zeta potential and (**G**) size of HAP, MNPs, and MHAP NPs measured by the DLS method.

The HAP NPs were synthesized by the wet chemical method. Initially, Ca(OH)_2_ (0.5 M) was mixed in double-distilled water (100 mL) at 90 °C. Then, while the solution was being vigorously stirred, an 0.3 M aqueous solution of H_3_PO_4_ was added slowly using a burette at a rate of 10 mL min^−1^. After 24 h of aging, the milky white precipitates were collected using the filter press, washed, and dried at 80 °C [15]. Later, to synthesize the MHAP NPs, the as-obtained HAP nanoparticles were added to the polyvinyl pyrrolidone (PVP) solution to form an interfacial binder between the surface of the HAP and MNPs formed by the co-precipitation of the Fe^3+^ and Fe^2+^ precursor solution (Figure 1A), and this process was followed by the MNPs formation. APTES was used as a capping agent to prevent oxidation and enhance the stability of nanoparticles [39]. From the Bio TEM image (Figure 1B), the synthesized HAP showed evidence for the homogenous-sized rod-shaped NPs which were 30 ± 2 nm in width and 120 ± 7 nm in length, and the EDS elemental mapping (Figure 1C) of HAP NPs confirmed the Ca and P signals. DLS results of HAP NPs, MNPs, and MHAP NPs showed an average particle size (Figure 1G) of 600 ± 39 nm, 165 ± 45 nm, and 480 ± 40 nm with an average zeta potential (Figure 1F) of −14.11 ± 7.57 mV, 30.93 ± 4.75 mV, and −5.99 ± 3.39 mV, respectively. Compared to TEM results, DLS results showed the increased size of the nanoparticle, which could be due to the aggregated HAP nanorods in the solution, could have increased the ensemble hydrodynamic diameter [40]. Meanwhile, MHAP NPs showed reduced hydrodynamic diameter compared to HAP NPs, which could be due to the better dispersion and steric stability achieved by APTES functionalization [41]. A similar hydrodynamic size reduction was recently noted because of APTES surface modification [42,43]. The zeta potential results evidenced the negatively charged HAP NPs, which could be correlated to early published reports that the HAP nanoparticles have evidenced a negative surface in the range of −7.3 to −17.7 mV [44]. The high positive charge of MNPs was due to APTES functionalization [45], and MHAP NPs negative surface charge was reduced compared to HAP NPs, due to the positive charge exhibited by MNPs. In this work, rod-shaped HAP nanoparticle was taken into consideration because natural bone is an inorganic material with nanocrystalline rod-like structures. Additionally, rod-shaped nanoparticles could provide better interaction via Van der Waals forces due to their higher superficial area. It has also been reported that rod-shaped HAP nanoparticles have high cell internalization rates and more prolonged blood circulation than spherical nanoparticles [46]. Similarly, Figure 1D,E showed the formation of MNPs on the surface of HAP NPs, and the EDS elemental mapping of MHAP NPs showed the Fe signals on the surface of Ca and P signals. The elemental composition of the composite MHAP NPs is identified as follows: O (59.16%), P (10.82%), Ca (22.37%), and Fe (7.65%).

**Figure 2:**
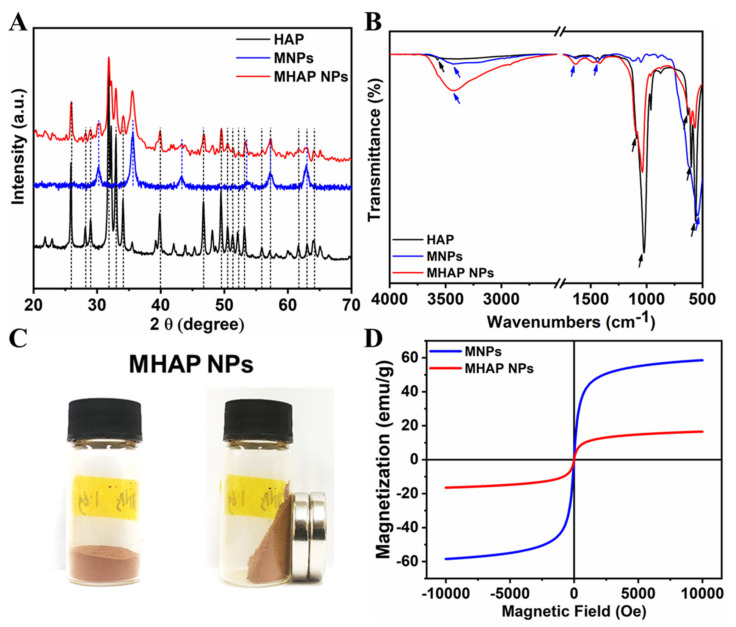
(**A**) XRD and (**B**) FTIR of synthesized HAP, MNPs, and MHAP NPs. (**C**) Digital images of the MHAP NPs show magnetic response to a static magnetic field using a mini magnet. (**D**) Field-dependent magnetization of MNPs and MHAP NPs.

The XRD pattern (Figure 2A) evidenced that the synthesized HAP NPs have a single phase with a hexagonal crystal structure. Furthermore, all the diffraction peaks were indexed, corresponding to reported pure HAP phase JCPDS No. 09-0432 data [47]. The XRD of MHAP NPs was also analyzed, and it found that MHAP NPs retained all the peaks of hexagonal HAP NPs with an extra peak at 2θ = 30.2° and 35.5°, which corresponds to the miller index (2 2 0) and (3 1 1) of (Fe_3_O_4_) MNPs, respectively [48]. The other extra peaks of the MNPs in MHAP NPs were not visible due to the interference of HAP NPs. However, all the major diffraction peaks of pristine MNPs synthesized without HAP were noted, corresponding to the JCPDS NO. 19-0629, which reported the pure phase of MNPs [49]. Furthermore, the FTIR spectra of HAP, MNPs, and the composite MHAP NPs are shown in Figure 2B. The MHAP NPs exhibited the characteristic peaks of HAP at 567 cm^−1^ and 602 cm^−1^ (triply degenerated ν4 bending of O–P–O bonds); 962 cm^−1^ (symmetric nondegenerate ν1 stretching mode of P–O); 1037 cm^−1^ and 1092 cm^−1^ (antisymmetric triply degenerate ν3 stretching vibration of PO_4_^3−^); 634 cm^−1^ (librational νL (O–H)); 3575 cm^−1^ (stretching vibration of νS (O-H)) [50] and characteristic peaks of MNPs at 3445 cm^−1^ (O–H stretching vibration); 1632 cm^−1^ (O–Hbending vibration); and 544 cm^−1^ (Fe-O stretching vibration), respectively [51]. Furthermore, APTES N-H bending and stretching modes of the terminal primary amine group could not be noted due to the peak overlap hydroxyl group stretching vibration at 3445 cm^−1^. However, the APTES grafting could be confirmed with the presence of C–H bond stretching vibrational peaks at 2851 cm^−1^ and 2927 cm^−1^, corresponding to the propyl group [52]. The magnetic properties of the synthesized MHAP NPs were noticed (Figure 2C) via a digital image of a magnetic response to a static magnetic field using a mini-round magnet bar. Furthermore, the magnetic properties of the MNPs and MHAP NPs were analyzed using a VSM (Figure 2D), and the results evidenced that MNPs and MHAP NPs exhibited saturation magnetization values of 58.53 and 16.49 emu/g, respectively. The decrease in MHAP NPs saturation magnetization to the pristine MNPs was due to the reduction in the volume of the magnetic component in the composite MHAP·NPs. Both samples exhibited negligible coercive field and remanence, as evidenced by the absence of hysteresis in the variation of magnetization for the applied field, which confirms the superparamagnetic features [53,54].

### 2.2. MHAP NPs Induced Cell Viability and Morphology

**Figure 3:**
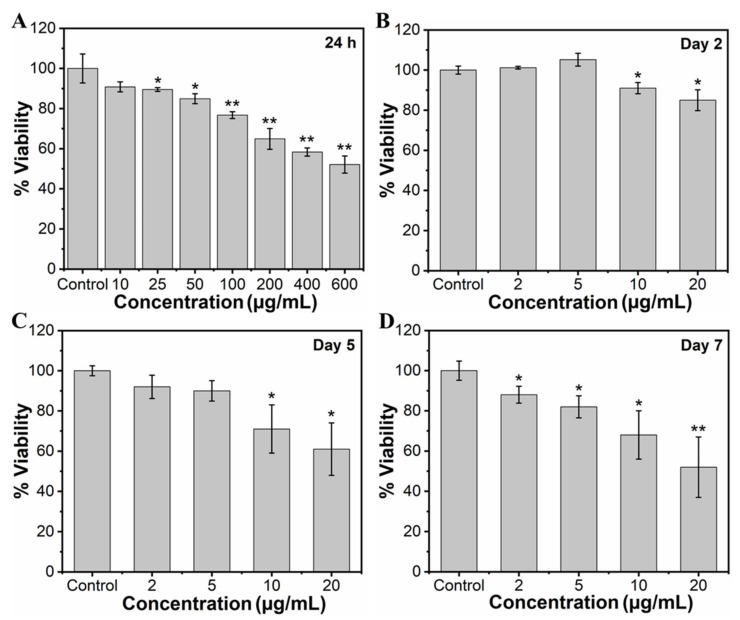
(**A**) Concentration-dependent (10–600 µg/mL) effect of MHAP NPs on cell viability of MC3T3-E1 cells for 24 h and the time-dependent effect of MHAP NPs on the viability of MC3T3-E1 cells. In addition, a single dose of NPs was treated with cells, and the viability of cells was analyzed on (**B**) day 2, (**C**) day 5, and (**D**) day 7, respectively, using MTT assay. Data were presented as mean ± standard deviation (*n* = 4) and significance (* *p* < 0.05; ** *p* < 0.01).

To study the effect of MHAP NPs on the viability of MC3T3-E1 cells, the cells were cultured with wide concentrations of NPs (10, 25, 50, 100, 200, 400, and 600 μg/mL) for 24 h. The viability of the cells was assessed through an MTT assay, as shown in Figure 3A. Considering a control exhibiting 100% cell viability, MHAP NPs samples with the concentrations of 10, 25, 50, 100, 200, 400, and 600 µg/mL exhibited approximately 90.81%, 89.50%, 84.91%, 76.72%, 64.91%, 58.36%, and 52.13% of cell viability, respectively, for 24 h. The results evidenced a significant increase in cytotoxicity with an increasing concentration of MHAP NPs. Cell viability was significantly reduced by ≈23.28% at 100 μg/mL and ≈47.87% at 600 μg MHAP NPs/mL. However, at an initial concentration of 10 µg/mL, no significant cytotoxicity was noted for 24 h of NPs treatment. Following the wider NPs treatment concentrations, we have narrowed down concentrations of MHAP NPs for long-term viability assessment. Thus, after narrowing down the concentration from 0 to 20 µg/mL of MHAP NPs, the cell viability result was noted as 93.30%, 90.07%, 88.0%, and 83.05% for 2, 5, 10, and 20 µg/mL, respectively, following the 24 h of MHAP NPs treatment (Figure S1). These findings could be correlated with the cytotoxicity results of MNPs reported earlier, which demonstrated that irrespective of MNPs size, increased cytotoxicity was noted at concentrations above 50 μg/mL [55]. Furthermore, the viability studies performed on the second day, fifth day, and seventh day were evidenced by cell viabilities of 74%, 61%, and 52% for 20 µg/mL of MHAP NPs (Figure 3B–D), respectively. The results indicated that the viability of preosteoblast MC3T3-E1 cells was significantly inhibited dose-dependently with time. In particular, 10 and 20 μg/mL MHAP NPs showed significant inhibition of mitochondrial function compared to the control. These results could be related to the lactate dehydrogenase (LDH; stable cytosolic enzyme from mitochondria released during cell lysis) cytotoxicity assay reported by Wu Hsi-Chin et al., who cocultured magnetic HAP NPs with rat-derived MSCs [56]. However, low concentrations (2 and 5 μg/mL) did not affect the viability.

**Figure 4:**
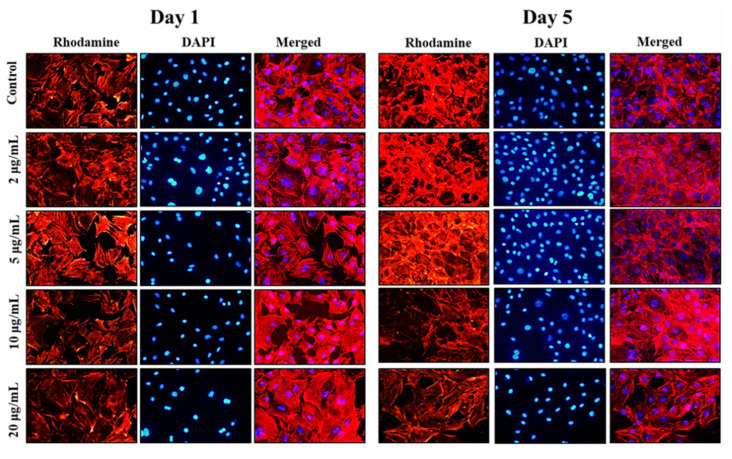
The inverted fluorescence microscope images show the morphological staining of MC3T3-E1 cells after being treated with MHAP NPs with varying concentrations (0–20 µg/mL) on days 1 and 5, respectively. The cytoplasm was stained with rhodamine-phalloidin (red), and nuclei were stained with DAPI (blue). The images were acquired at 20× magnification.

Following the cell viability results, inverted fluorescence microscope imaging was carried out to provide evidence for the morphological changes of the preosteoblast cells following the treatment with NPs on days 1 and 5 using rhodamine-phalloidin (cytoskeleton) and DAPI (nuclei) staining. The results of inverted fluorescence microscope images (Figure 4) showed no apparent changes in the cytoskeletal architecture of MC3T3-E1 cells after being cocultured with nanoparticles. Compared to the control cells, the cells treated with the 5 and 10 µg/mL of MHAP NPs evidenced slightly more elongation on day 5 compared to day 1. These results indicate that cell proliferation and expanded focal adhesion over the MHAP NPs treatment. A clustered and confluent morphology has also been evidenced with adjacent cells. The findings are consistent with MTT cell viability studies. Similar topology and architecture on HA/Ca/Mg scaffolds were also seen in prior research. Our results are consistent with those made in earlier research on HA/Ca/Mg scaffold reported by Chu et al., in which a similar morphology was shown [57].

### 2.3. MHAP NPs Induced ROS Production and Apoptosis

**Figure 5:**
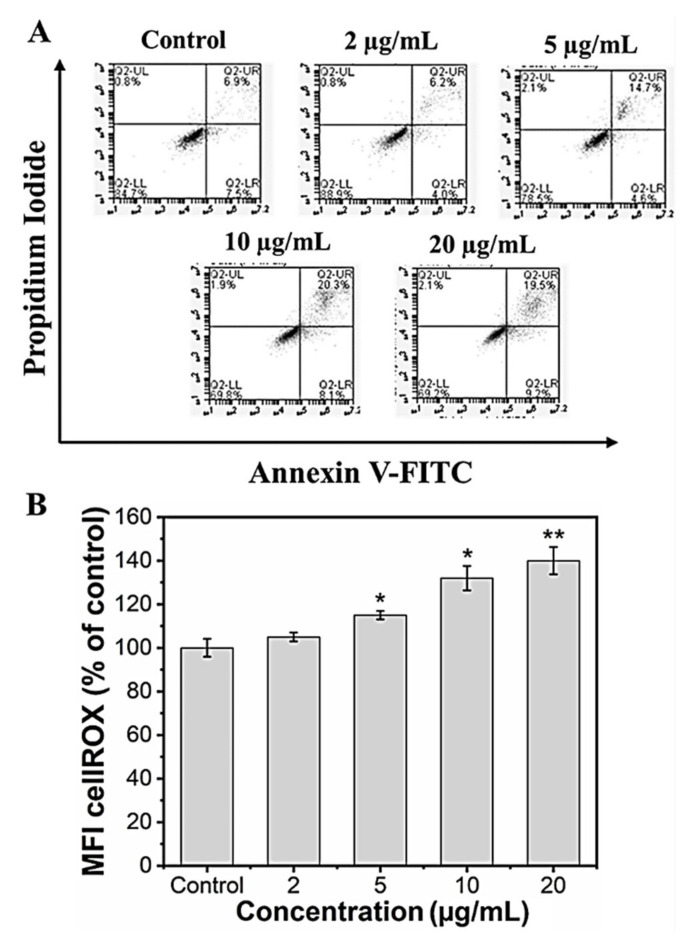
(**A**) Live and dead apoptosis FACS analysis; (**B**) mean fluorescence intensity (MFI) of CellROX of MC3T3-E1 cells. (Data were presented as mean ± standard deviation (*n* = 4) and significance (* *p* < 0.05; ** *p* < 0.01).

It is known that nanoparticles could induce cell death via necrosis or apoptosis. Necrosis is associated with extensive damage and intense inflammatory response, whereas apoptosis is programmed cell death [58]. In our in vitro study, we checked whether the reduced viability in the preosteoblast cells following MHAP NPs treatment was not due to apoptotic induction. We performed an annexin V/Propidium iodide assay to assess the apoptotic cell population following the MHAP NPs treatment for 24 h. The results indicated that the cells that were treated with a lower concentration (2 μg/mL) of MHAP NPs showed evidence for cell viability of 90.1 ± 3.2% when compared to the control (85.3 ± 1.6%). However, when the concentrations exceeded 10 μg/mL (70 ± 2.5%), it caused significant apoptosis, as shown in (Figure 5A). These FACS results were also in correlation with the MTT assay cell viability studies. A previous survey of HAP nanoparticles reported that the NPs could induce cell death at a concentration above 10 μg/mL in MC3T3 cells, which is in line with our current findings [59].

Reactive oxygen species (ROS) are secondary products generated during oxygen utilization and cellular metabolism. ROS production at low levels can support cell proliferation and differentiation; in contrast, high levels of ROS generation lead to apoptosis [60]. The mean fluorescence intensity of CellROX was increased to 105.2 ± 2%, 110.7 ± 1.9%, 132.4 ± 8.2%, and 140.5 ± 7.3% for the concentrations 2, 5, 10, and 20 µg/mL of MHAP NPs compared to control (100 ± 4.1%) after 24 h. These results indicate that MHAP NPs at an increasing concentration significantly enhanced ROS production (Figure 5B). However, the ROS production was in a low concentration for 2 and 5 µg/mL of MHAP NPs compared to 20 µg/mL, which could also aid in low ROS-induced osteogenic differentiation of MC3T3-E1 cells. Recent research showed that MNPs produced ROS after internalization into the cells and orchestrated various signaling pathways [61]. The size of the nanoparticle is critical in inducing ROS production and cytotoxicity; smaller NPs <100 nm were shown to have enhanced toxicity compared to large-sized particles [62]. The synthesized MHAP NPs are <100 nm in size in the present study.

### 2.4. Effect of MHAP NPs on Osteoblast Differentiation

**Figure 6:**
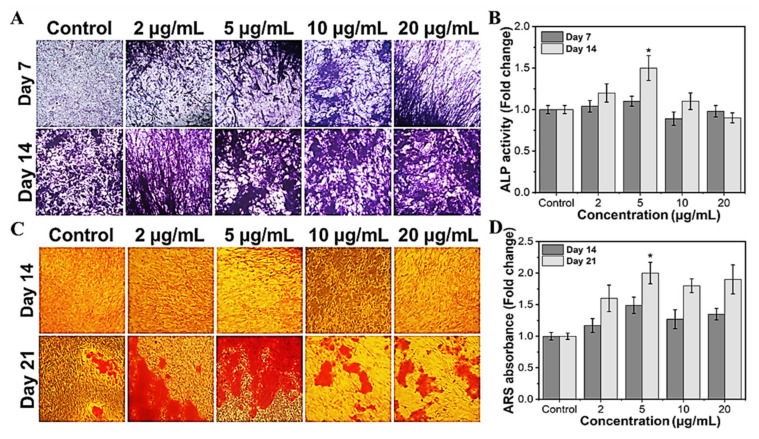
Osteogenic differentiation potential of MHAP NPs with different concentrations. (**A**) The optical microscopic images of ALP-stained samples, and (**B**) the ALP enzymatic activity of MC3T3-E1 cells on days 7 and 14. (**C**) The optical microscopic image of ARS stained samples, and (**D**) ARS calcium quantification of MC3T3-E1 cells on days 14 and 21. Data were presented as mean ± standard deviation (*n* = 4) and significance (* *p* < 0.05).

To investigate the efficacy of MHAP NPs in osteoblast differentiation, we evaluated the ALP and calcification, which are known as phenotypic markers of osteoblast differentiation. Trace elements are required to function in regular biological processes in living systems. Specifically, Fe and Zn are regarded as essential components. They serve as cofactors in several metabolic reactions, and their concentrations are tightly regulated to ensure homeostasis [63]. In the current study, we investigated the potential of the MHAP NPs to promote bone differentiation, which could also modulate the level of ROS within the cell, which in turn alters the activity of the enzymes and could encourage the deposition of calcium on the cell surface’s matrix. The MHAP NPs treatments at less than 10 μg/mL significantly increased the ALP activity (Figure 6A,B) compared to the untreated control on day 14. In MC3T3-E1 cells, elevated ALP enzyme activity is considered characteristic of the initial stage of osteoblast development [64]. The outcomes are highly correlated and in agreement with those of the control samples. It is clear from prior research that less ROS impacts the expression of genes related to bone development, including ALP expression [65]. In contrast to the ALP assay, the calcification (Alizarin red staining) is considerably higher in all the treated concentrations (Figure 6C,D). MC3T3-E1 cells cultured with MHAP NPs (all concentrations) had more calcium deposition than the control. After 21 days of incubation with various concentrations of MHAP NPs, 5 μg/mL treatment was found to be optimal in the ALP and calcification assay. Thus, the current study evidenced that the synergistic effect of MHAP NPs (5 μg/mL), even at a low concentration, could promote osteogenic differentiation and adequate calcium mineralization for bone tissue reconstruction. Supporting the above results, HAP and MNPs are known for physical and biochemical stimuli, which elicit chromosomal responses which regulate the mitogen-activated protein kinase (MAPK) pathway, which in turn leads to upregulation of ALP, BMP2, and Smad proteins, resulting in the expression of RUNX2, an early osteogenic differentiation marker that plays a vital role in multiple major signaling pathways that promote osteogenesis [66,67,68,69].

## 3. Materials and Methods

### 3.1. Materials

The materials used in the present study include scallop-shell-derived calcium hydroxide (Ca(OH)_2_ ≥ 96%, Natural Japan Co Ltd., Hokkaido, Japan), phosphoric acid (H_3_PO_4_, 85%, Sigma-Aldrich, St. Louis, MO, USA), iron(III) chloride hexahydrate (97%, Sigma-Aldrich, St. Louis, MO, USA), iron(II) sulfate heptahydrate (≥ 99%, Sigma-Aldrich, St. Louis, MO, USA), sodium hydroxide (Samchun chemicals, Pyeongtaek, Republic of Korea), 3-aminopropyltriethoxysilane (APTES, 98%, Sigma-Aldrich, St. Louis, MO, USA), MC3T3-E1 (ATCC^®^ CRL-2593^™^, Seoul, Republic of Korea). 

### 3.2. Preparation of Hydroxyapatite Nanoparticles (HAP NPs)

As reported earlier, the precipitation technique synthesized the HAP nanoparticles in an aqueous system with a slight modification [15]. Briefly, calcium hydroxide (0.5 M, 100 mL) was mixed in DI water and maintained at 90 °C. After 1 h, phosphoric acid (0.3 M, 100 mL) was added dropwise into the aqueous hydroxide solution at a rate of 10 mL min^−1^. White precipitates formed were allowed 24 h of aging and collected by centrifugation (at 7000 rpm for 10 min, Fleta, Hanil, Gimpo, Republic of Korea). After washing 5 times with excess water, the collected particles were dried for 24 h at 80 °C to obtain crystalline HAP nanoparticles.

### 3.3. Preparation of Magnetic Nanoparticle Decorated HAP NPs (MHAP NPs)

The MHAP nanoparticles were synthesized by the co-precipitation wet chemical method. Briefly, 1 g of HAP nanoparticles was dispersed in the mixture of 1.5 mM FeSO_4_·7H_2_O and 3 mM FeCl_3_·6H_2_O aqueous solution. Followed by sonication for 30 min, the mixture was stirred at 70 °C with N_2_ bubbling for 30 min, and then 3M sodium hydroxide was added to the reaction mixture to adjust the pH 11. After 40 min, 150 µL of APTES (250 mg/mL) is added as a capping agent and continued the reaction for 1 h [70]. Finally, the obtained MHAP precipitates were centrifuged, washed several times, and dried at 60 °C for 24 h, followed by mortar and pestled to obtain finely granulated nanoparticles.

### 3.4. Physicochemical Characterization

The particle size and morphology of HAP and MHAP NPs were analyzed using a Field Emission Scanning Electron Microscope (FESEM, ZEISS SUPRA 40VP). Additionally, the size and the surface charge of HAP, MNPs, and MHAP NPs were analyzed using a Zetasizer instrument (NanoZ590, Malvern Instruments, Worcestershire, UK). The Energy Dispersive X-ray Spectroscopy (EDS) elemental mapping and composition analysis were also performed with FESEM. TEM images were acquired by Transmission Electron Microscopy (TEM, JEOL, JEM-2010). The crystallinity of HAP and MHAP NPs were analyzed using X-ray powder diffraction (XRD, PANalytical X′pert Pro Powder, Worcestershire, UK) with monochromatic Cu Kα (λ = 1.5405 Å) over the range of Bragg angle (10–80°). Fourier transform infrared spectroscopy (FT-IR, Perkin Elmer Frontier, Waltham, MA, USA) analysis was performed at a frequency range of 4000–500 cm^−1^ to confirm the characteristic spectra of synthesized HAP and MHAP nanoparticles. Magnetic characterization was performed on a vibrating sample magnetometer (8600 Series VSM, Lake Shore Cryotronics, Inc., Westerville, OH, USA). The magnetic hysteresis loop measurement was performed at room temperature under external magnetic fields from −10 to 10 KOe.

### 3.5. Viability Assay

The preosteoblast MC3T3-E1 cells were cultured in alpha MEM medium supplemented with heat-inactivated 10% fetal bovine serum (FBS) and 1% antibiotic-penicillin/streptomycin solution and incubated at 37 ℃ with 5% CO_2_ in a humidified incubator. Briefly, in a 96-well plate, 5 × 10^3^ cells per well were seeded and incubated for 12 h. Followed by the incubation, different concentrations (10, 25, 50, 100, 200, 400, and 600 µg/mL) of MHAP NPs were added into respective wells. After 12 h MHAP NPs treatment, the excess NPs were carefully removed with PBS wash. Meanwhile, NPs untreated wells served as the control. After 24 h of MHAP NPs treatment, the cytotoxicity was evaluated using cell proliferation kit I (MTT) (Sigma-Aldrich, St. Louis, MO, USA). In short, MTT (3-(4,5-dimethylthiazol-2-yl)-2,5 diphenyl tetrazolium bromide) was added to the cells at a final concentration of 0.5 mg/mL and incubated for 4 h to form insoluble formazan crystals. Later, using a solubilizing solution from the kit, the formazan was solubilized and analyzed using a microplate reader at 570 nm absorbance [71,72].

The proliferation study of the preosteoblast MC3T3-E1 cells, which was followed by the cytotoxicity assay, was analyzed within the limit of 20% inhibitory concentrations (IC20) with different concentrations (2, 5, 10, and 20 µg/mL) of MHAP NPs. Briefly, for the proliferation assay, after 24 h of MHAP NPs addition, the culture medium was changed once, then it was changed every two days once the culture medium was replaced with fresh medium. On the 2nd, 5th, and 7th day, the MTT assay was performed as mentioned above. All the experiment was replicated three times and plotted.

### 3.6. Apoptosis Analysis

The preosteoblast MC3T3-E1 cells were treated with different concentrations of MHAP NPs (0–20 µg/mL) for 24 h and were washed with PBS. Later, the cells were trypsinized and collected by centrifugation and stained using the FITC Annexin V apoptosis detection kit (BD Biosciences) according to the manufacturer’s instruction and analyzed using a flow cytometer (BD Accuri C6 cytometer) [73,74].

### 3.7. ROS Production

Reactive oxygen species (ROS) production following the MHAP NPs treatment was analyzed with an ROS detection kit with CellROX Green reagent (ThermoFisher, Waltham, MA, USA) according to the manufacturer’s instructions. Briefly, 1 × 10^4^ MC3T3-E1 cells were seeded into a 48 well-plate and incubated with various concentrations of MHAP NPs (2, 5, 10, 20 μg/mL) for 24 h. Following the incubation, the cells were trypsinized, washed with PBS, and incubated with 5 μM CellROX Green reagent for 60 min at 37 °C in the dark. After washing with PBS, fluorescence was measured immediately using flow cytometry (BD Accuri) [75].

### 3.8. Morphological Analysis

To study any morphological changes induced by MHAP NPs, the MC3T3-E1 cells were treated with various concentrations (2, 5, 10, and 20 µg/mL) of NPs, and the cells’ morphological analyses were conducted on days 1 and 5. After incubation with NPs, the cells were washed with PBS and fixed using 4% paraformaldehyde for 10 min at room temperature. Next, the fixed cells were permeabilized using 0.1% Triton-X 100 in PBS for 5 min. Later, the cells were blocked with 0.5% Bovine serum albumin (BSA) for 15 min, incubated with rhodamine-phalloidin for actin filament staining (30 min), and washed with PBS. Following the incubation, the cells were stained for nuclei using DAPI (4′,6-diamidino-2-phenylindole) fluorescence staining for 5 min at RT in the dark, and the cells were washed with PBS. Finally, the fluorescence signals were measured using an inverted fluorescence microscope [76]. The images were acquired at a magnification of 20× from three different spots on the stained samples.

### 3.9. Alkaline Phosphatase Assay

Alkaline phosphatase levels following MHAP NPs treatment in MC3T3-E1 cells were analyzed using an alkaline phosphatase detection kit (Millipore) as per the manufacturer’s instructions. Briefly, cells were seeded into a 48 well-plate; once the growth confluence reached above 80%, the medium was replaced with osteogenic induction medium (OIM) containing 10 mM β-Glycerol phosphate (Sigma-Aldrich, St. Louis, MO, USA) and 50 μg/mL ascorbic acid in complete growth medium. Every 2 days once, the OIM was replaced until the analysis. On days 7 and 14, all the samples were rinsed with PBS several times before being fixed in the 4% paraformaldehyde, and the samples were displayed for 30 min and incubated at 37 °C for 15 min, followed by adding 330 µL BCIP/NBT solution. Then, the staining sample could be observed after washing, and the images were acquired at a magnification of 20× from three different spots for each sample. Furthermore, a quantitative analysis of ALP secretion was performed through an alkaline phosphatase assay kit. Briefly, the protein samples were collected by RIPA lysis buffer, and then the total protein volume was detected with a BCA Protein Assay Kit (ThermoFisher, Seoul, Republic of Korea). The ALP secretion was expressed by the fold change in the OD value of ALP/total proteins [77].

### 3.10. Calcium Deposition Assay (Alizarin Red Staining)

Alizarin staining kit was used to observe the calcium deposition in MC3T3-E1 cells after adding various concentrations (2, 5, 10, and 20 μg/mL) of MHAP NPs. The cells were cultured in OIM, and the medium was changed every 2 days until analysis. On day 21, the cells were washed with phosphate-buffered saline and fixed with 4% paraformaldehyde at room temperature. Later, the fixed cells were washed thrice with distilled water and treated with an alizarin red staining solution for 30 min, followed by two distilled water washes, and the cells were air-dried. The calcium nodules in each condition were observed using an inverted microscope, and the images were acquired at a magnification of 20× from three different spots on the cell culture plates for each sample. The cells stained with ARS were subjected to cetylpyridinium chloride digestion, and the absorbance was measured at 540 nm using spectrophotometry according to the published methodology, with minor alterations [78].

### 3.11. Statistical Analysis

One-way analysis of variance and a post-hoc Tukey comparison test with a 95% confidence level was used for all statistical analyses, with mean ± standard deviation (S.D., *n* = 3 (* *p* < 0.05, ** *p* < 0.01, *** *p* < 0.001)).

## 4. Conclusions

Developing smart biomaterials could aid in regenerating osteoporotic bone in situ. Magnetic ferrite-based materials are gaining appeal in regenerative medicine for MRI contrasts, magnetically driven hyperthermia, drug delivery, and tissue regeneration. In this study, hydroxyapatite bone-mimicking bioceramics were decorated with magnetic nanoparticles using a simple wet chemical co-precipitation method, and their synergistic effect was investigated for cytotoxicity, proliferation, ROS activity, and osteogenic differentiation potentials in MC3T3-E1 cells. Furthermore, the concentration-dependent ROS activity was noted with potential osteogenic differentiation by facilitating ALP activity and calcium deposition compared to control cells. The in vitro results showed that an optimal concentration of 5 µg/mL of synthesized MHAP NPs showed no toxicity with higher osteogenic differentiating potentials. Therefore, the synthesized magnetic hydroxyapatite composite nanomaterial could potentially be applied in regenerative medicine, especially in bone tissue engineering. In the future, the developed magnetic hydroxyapatite nanoparticles could be employed to deliver magnetically triggered biomechanical cues in combination with 3D scaffolds and in vivo evaluation for bone tissue regeneration.

## Data Availability

The data presented in this study are available on request from the corresponding author.

## Supplementary Materials

The following supporting information can be downloaded at: https://www.mdpi.com/article/10.3390/md21020085/s1, Figure S1: MTT assay showing % of MC3T3-E1 cell viability treated with varying concentrations (0–20 µg/mL) of MHAP NPs for 24 h, respectively. Data were presented as mean ± standard deviation (*n* = 4) and significance print (* *p* < 0.05).

## Sample Availability

Samples of the compounds are available from the authors.
