# Supplementary material for: Magnetic Hydroxyapatite Composite Nanoparticles for Augmented Differentiation of MC3T3-E1 Cells for Bone Tissue Engineering
Source: Mar Drugs. 2023 Jan 25;21(2):85. doi: 10.3390/md21020085 (PMC9960960; doi:10.3390/md21020085)
Supplement: Supplementary file 1 [file marinedrugs-21-00085-s001.zip › marinedrugs-2145298-supplementary.pdf]

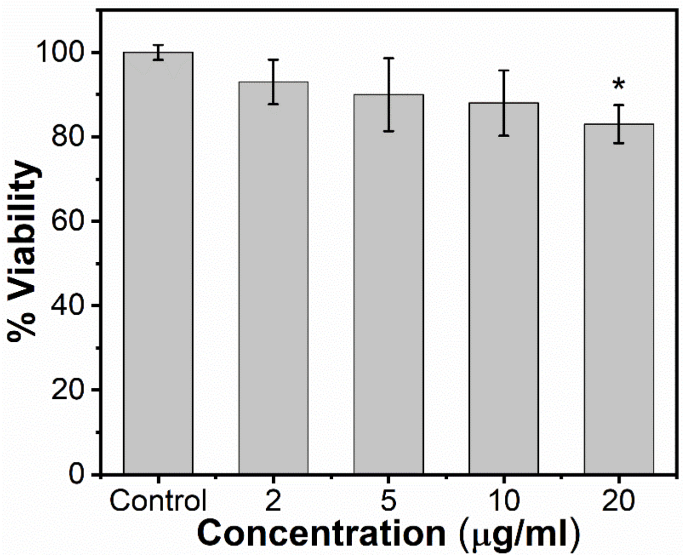

Figure S1. MTT assay showing % of MC3T3-E1 cell viability treated with varying concentrations (0-20 μg/ml) of MHAP NPs for 24 h, respectively. Data were presented as mean ± standard deviation (n=4) and significance print (*$p < 0.05$).
